# Ancient Origins of Allosteric Activation in a Ser/Thr Kinase

**DOI:** 10.1126/science.aay9959

**Published:** 2020-02-21

**Authors:** Adelajda Hadzipasic, Christopher Wilson, Vy Nguyen, Nadja Kern, Chansik Kim, Warintra Pitsawong, Janice Villali, Yuejiao Zheng, Dorothee Kern

**Affiliations:** Department of Biochemistry and Howard Hughes Medical Institute, Brandeis University, Waltham, MA 02454

## Abstract

Despite a myriad of cellular events being regulated by allostery, evolution of this process is largely unexplored territory. Here, we use Ancestral Sequence Reconstruction (ASR) to resurrect ancestors of two colocalizing proteins, Aurora A kinase and its allosteric activator TPX2, to experimentally characterize the evolutionary path of allosteric activation. Autophosphorylation of the activation loop is the most ancient activation mechanism; it is fully developed in the oldest kinase ancestor and remains stable over one billion years of evolution. As the microtubule-associated protein TPX2 appeared, efficient kinase binding to TPX2 evolved likely due to increased fitness by virtue of colocalization. Subsequently, TPX2-mediated allosteric kinase regulation gradually evolved. Surprisingly, evolution of this regulation is encoded in the kinase and did not arise by a dominating mechanism of coevolution.

## One Sentence Summary

Evolution of allosteric regulation is studied by Ancestral Sequence Reconstruction of Aurora kinase together with its activation partner TPX2.

**Fig. 1.**
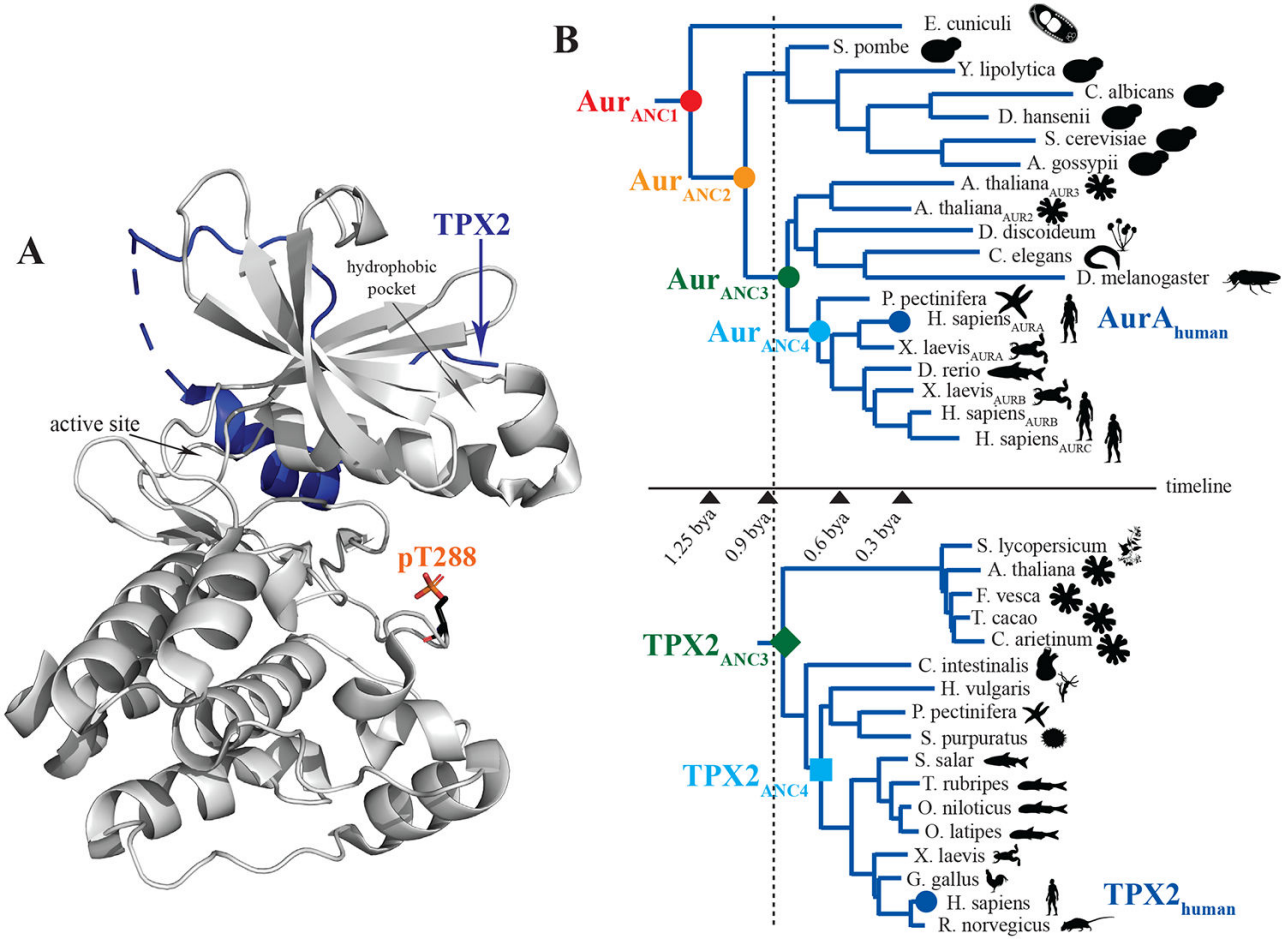
Strategy to study the ancient origins of allosteric activation. (A) X-ray structure of human Aurora A kinase (AurA) bound to TPX2 (PDB ID 1OL5) illustrating two distinct AurA allosteric activation mechanisms: binding of TPX2 to the AurA hydrophobic pocket or phosphorylation of activation loop at T288. (B) Phylogenetic trees for Aurora kinase and TPX2 calculated using BAli-Phy (12) are shown together with a rough time estimate, indicating that the older ancestors of Aurora existed before the emergence of TPX2. Color coding for all species defined here is used throughout the manuscript.

Allosteric regulation, the process by which a protein’s activity can be modulated by binding of an effector molecule distal to the active site, was first formalized over half a century ago by the groundbreaking work of Monod, Wyman and Changeux (1). Since then, allosteric regulation has been shown to be vital for cellular signaling processes ranging from oxygen transport to synaptic transmission to modulation of catalytic rates of enzymes (2-4). Given the central role of allosteric control, we wanted to experimentally explore how allosteric regulation developed along evolutionary pathways, a topic that has not been tackled before. We chose to examine the evolution of allosteric regulation for a modern Ser/Thr protein kinase since the vast majority of Ser/Thr kinases are regulated by two distinct allosteric mechanisms, phosphorylation and specific protein/protein interactions (Fig. 1A), thereby providing signaling diversity. We chose Aurora A, an essential kinase for the cell cycle – aberrant levels of Aurora A lead to improper centrosome maturation, spindle formation, mitotic entry and enhanced cancerous growth (5-7). Binding of TPX2 (Targeting Protein for Xklp2) to the hydrophobic pocket of Aurora A both targets Aurora A to the spindle microtubules and also allosterically activates the kinase (8, 9).

First proposed by Linus Pauling and Emile Zuckerkandl, ancestor sequence reconstruction (ASR) allows for the recreation of ancestral proteins in the laboratory so that inferred pathways of evolution can be studied directly (10, 11). By resurrecting ancestors of both Aurora A kinase and its activator TPX2 along the evolutionary path, we identify activation loop autophosphorylation as the oldest allosteric activation mechanism, followed by TPX2-regulation that evolved in a gradual fashion. The data expose the molecular mechanism of the evolving network in the kinase–allosteric activator pair and aid in defining the allosteric network in the modern kinase.

A Bayesian phylogeny and co-estimated alignment of representatives of all known Aurora kinases and the closely related AGC and CAMK serine/threonine protein kinases was constructed using the program package BAli-Phy (12). Interestingly, the resulting Aurora phylogentic tree has the kingdoms Animalia and Plantae adjacent to one another with Fungi more distantly related. This relationship does not match the widely accepted species tree nesting of Animalia and Fungi being more closely related to each other than to Plantae. Our Aurora kinase gene tree topology is not unique and has been observed in other inferred Aurora kinase gene phylogenies (13, 14). Discrepancies between gene trees and species trees are common and can be caused by several factors including unequal rates of evolution, gene duplication and loss or horizontal gene transfer (15-17). Subsequently, the maximum a posterior (MAP) phylogeny was used as the input for PAML (18) to infer the most probable ancestral sequences (Fig. 1B and Fig. S1A-C, E). Similarly, TPX2 homologues from various organisms were used to infer the TPX2 ancestor sequences (Fig. 1B and Fig. S1D). A comparison of both trees guided by the phylogeny of the organisms reveals that while Aurora kinases are ubiquitous throughout the Eukaryotic kingdom, TPX2 containing the AurA binding sequence is only present in certain plants and animal species, but not in Protista or Fungi (19). This suggests the appearance of TPX2 post-dates that of the Aurora family (Fig. 1B), and indicates that Fungi lost the Aurora binding part of TPX2. The latter point provides a possible explanation of why the Aurora gene tree has Plantae closer to Animalia than Fungi.

**Fig. 2.**
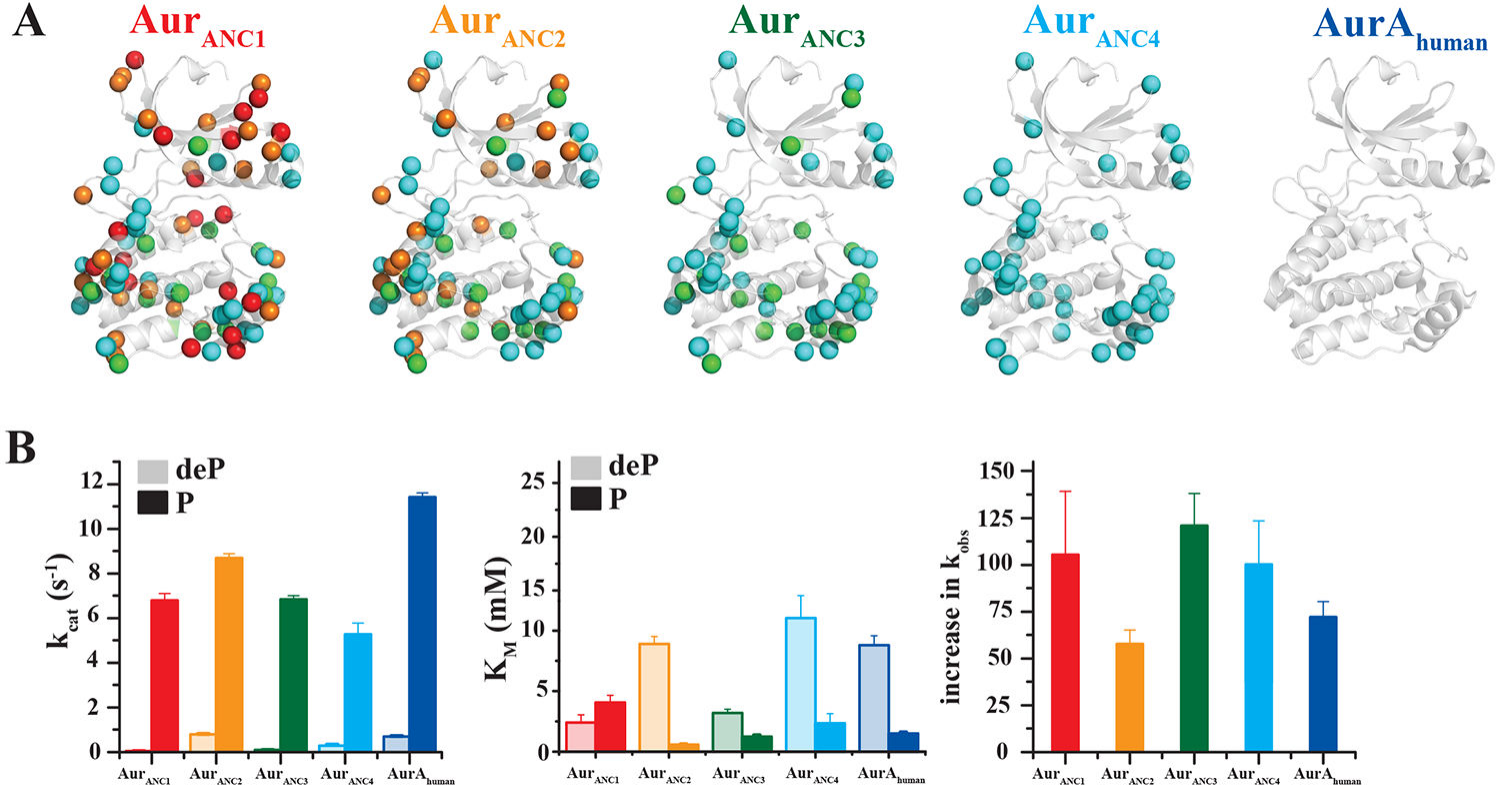
Autophosphorylation is the oldest and uniformly conserved allosteric activation mechanism in all Aurora kinases. (A) Additive differences in primary sequence between AurA_human_ and Aur_ANC4_ (82.4% identity), Aur_ANC3_ (80.1% identity), Aur_ANC2_ (72.7% identity) and Aur_ANC1_ (69.5% identity) are shown in cyan, green, orange and red spheres, respectively. (B) Corresponding k_cat_ (left) and K_M_ (middle) for dephosphorylated (deP) and T288-phosphorylated (P) Aurora kinases show that phosphorylation increases catalytic efficiency by increasing k_cat_ and decreasing K_M_ (see also Fig. S4). To best illustrate this combined effect, the fold increase in k_obs_ at 1 mM Lats2 upon Aurora’s T288 phosphorylation is shown on the right. Phosphorylation of Lats2 peptide was monitored using the ADP/NADH coupled assay with 1 μM dephosphorylated or 0.05 μM phosphorylated Aurora and 5 mM ATP, 20 mM MgCl_2_ at 25°C. Error bars represent the standard error for the estimate of k_cat_ or K_M_ through the Michaelis-Menten equation and are a measure of the goodness of fit of the data. Error bars for increase in k_obs_ are calculated using jackknifing and error propagation.

Two Aurora kinase ancestor sequences (Aur_ANC1_ and Aur_ANC2_) from an evolutionary period prior to the appearance of a canonical TPX2 motif, and two additional ancestor sequences (Aur_ANC3_ and Aur_ANC4_) with their corresponding TPX2 ancestor sequences (TPX2_ANC3_ and TPX2_ANC4_) (Fig. 1B and Fig. S2) were expressed and functionally characterized. Due to the later appearance of TPX2, we hypothesized that activation-loop autophosphorylation may have evolved first. Enzyme activity for dephosphorylated and phosphorylated Aurora forms was measured by monitoring phosphorylation of a peptide derived from the natural Aurora A kinase substrate Lats2 (Large tumor suppressor kinase 2, (5) (Fig. 2 and Fig. S3,4). All four ancestor Aurora kinases had very low activity in their dephosphorylated form. Importantly, all were activated by autophosphorylation of T288 to a similar extent (Fig. 2B). Surprisingly, T288-phosphorylation resulted not only in higher catalytic rates, but we also observed the evolution of additional regulation via tightening of the substrate K_M_ upon phosphorylation starting with Aur_ANC2_ (Fig. 2B). The combination of both effects, increase in k_cat_ and decrease in K_M_ upon phosphorylation, yields an increased catalytic efficiency of more than 50- fold upon activation loop phosphorylation for all Aurora forms tested (Fig. 2B).

**Fig. 3.**
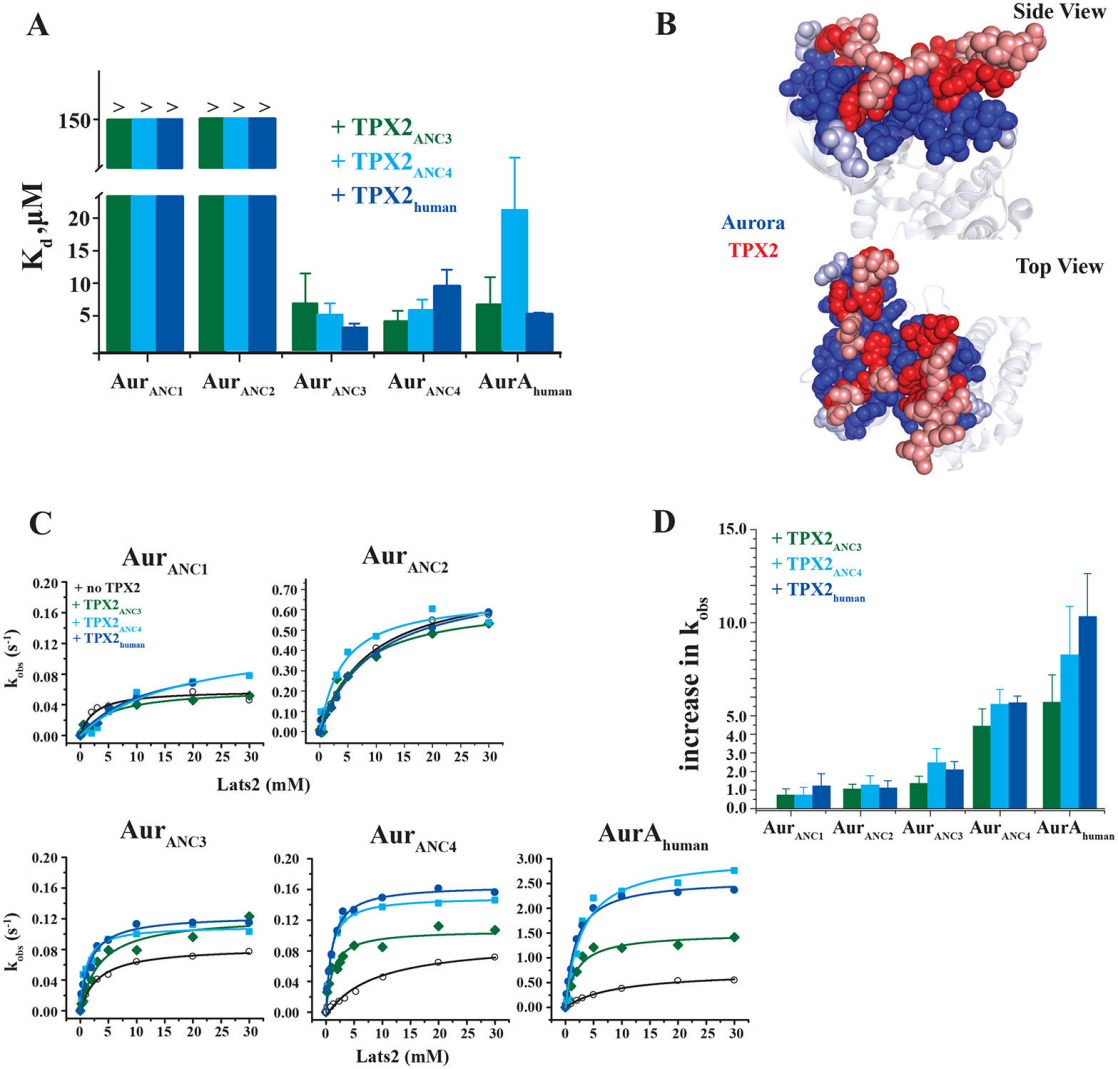
Allosteric activation via TPX2 binding is a more recent, gradually dialed in, regulatory adaptation for Aurora kinases. (A) Dissociation constants (K_d_) of ancestral and modern TPX2 constructs binding to all Aurora kinases measured by ITC. (B) Conservation of the Aurora (blue)/TPX2 (red) interface residues. Evolutionarily conserved residues in Aur_ANC3_, Aur_ANC4_ and AurA_human_ (dark blue) and in TPX2_ANC3_, TPX2_ANC4_ or TPX2_human_ (dark red) are plotted onto the interface zoom-in of the human Aurora A/TPX2 X-ray structure (PDB ID 1OL5) highlighting interface residues that are within 5Å in van der Waal’s contacts (non-conserved residues are in light blue and light red). (C) Michaelis-Menten plots of ancestral and human Aurora in the absence and presence of TPX2_ANC3_, TPX2_ANC4_ or TPX2_human_. 100μM TPX2 (for Aur_ANC3_, Aur_ANC4_ or AurA_human_ assuring saturation) and 500μM TPX2 (for Aur_ANC1_ or Aur_ANC2_) were used. (D) Gradual evolution of allosteric Aurora kinase activation by TPX2 binding illustrated as fold increase in k_obs_ at 1 mM Lats2. Activity assays were carried out as described above. Error bars in (A) represent the standard error for the estimate of K_d_ from the isotherms and are a measure of the goodness of fit of the data. Error bars for increase in k_obs_ in (D) are calculated using jackknifing and error propagation.

How did the second mode of allosteric modulation by the activator TPX2 evolve? According to the widely held view of coevolution as “reciprocal evolutionary change in interacting species” (20), we might expect that only the evolutionarily matching partners display allosteric activation. For allosteric activation by a second protein, two events need to evolve: physical binding, and an increase in kinase activity upon activator binding. We find that Aurora ancestors from the era prior to the appearance of TPX2 (Aur_ANC1_ and Aur_ANC2_) bind very weakly to TPX2s (Fig. 3A and Fig. S5), in agreement with our temporal matching of the two evolutionary trees. As soon as TPX2 became available, tight binding to Aurora was established. However, all of the more recently evolved Aurora ancestors (Aur_ANC3_ and Aur_ANC4_) including AurA_human_ bind all TPX2s with similar affinities, and not only their evolutionarily matching TPX2 partners (Fig. 3A and Fig. S5). This suggests that efficient TPX2 binding to Aurora was established as soon as TPX2 appeared and remained stable throughout evolution. In fact, the Aurora-TPX2 interaction surface remained extremely conserved starting from its initial appearance in Aur_ANC3_ and TPX2_ANC3_ to the human species (Fig. 3B and Fig. S2). We speculate that binding alone added an immediate evolutionary advantage by virtue of colocalization and delivery of the kinase to the spindle microtubules.

Besides this first act of increased fitness due to binding, how did allosteric activation by TPX2 evolve? Measuring the changes in kinase activity for Aur_ANC3_, Aur_ANC4_ and AurA_human_ in the presence of the matching TPX2-partners TPX2_ANC3_, TPX2_ANC4_ and TPX2_human_, respectively, a gradual increase in allosteric activation is observed (2x **→** 5x **→** 10x increase in enzymatic rate in the presence of TPX2_ANC3_, TPX2_ANC4_ and TPX2_human_, respectively) (Fig. 3C, D). Crucially, this gradual allosteric activation is predominantly encoded in the evolution of the kinase since the “mismatched” TPX2 forms also create this effect. Only a small additional increase in k_obs_ is measured for TPX2_ANC4_ and TPX2_human_ relative to older TPX2_ANC3_ in activating Aur_ANC4_ and AurA_human_ (Fig. 3C, D). To test the robustness of the ancestral reconstructions for these key findings, we sampled alternates from the ancestral posterior probability distribution for the key nodes Aur_ANC2_ and Aur_ANC3_ (Fig. S6) (21). Activity, binding and allosteric activation by TPX2 for these alternates buttress our conclusions (Fig. S7).

Since our data do not support a dominating mechanism of reciprocal change in coevolution of allosteric activation, we felt the need for additional tests for our new model and turned to Aurora B (AurB_human_), a close, allosterically activated homologous protein to Aurora A. First, AurB_human_’s allosteric activation partner INCENP that has no similarity to TPX2, can activate Aurora A by the same amount as TPX2, although INCENP binding is weaker (Fig. S8). That means that evolution of specificity between Auroras and their corresponding allosteric partners happened on the level of affinity, and the origin of allosteric activation is indeed encoded in the kinase with almost no reciprocal changes occurring in TPX2 to facilitate increased allosteric activation. Second, coevolution between the kinase and the substrate was ruled out by repeating all activity experiments with a second, generic substrate, kemptide. The allosteric activation measured using kemptide is within experimental error of that measured using the natural substrate Lats2 (Fig. S9). Third, none of the TPX2s allosterically increased the activity of Aurora ancestors from the pre-canonical-TPX2 era (Aur_ANC1_ and Aur_ANC2_) even at very high TPX2 concentrations where a substantial fraction of these Aurora ancestors is in the TPX2-bound states (Fig. 3C,D).

**Fig. 4.**
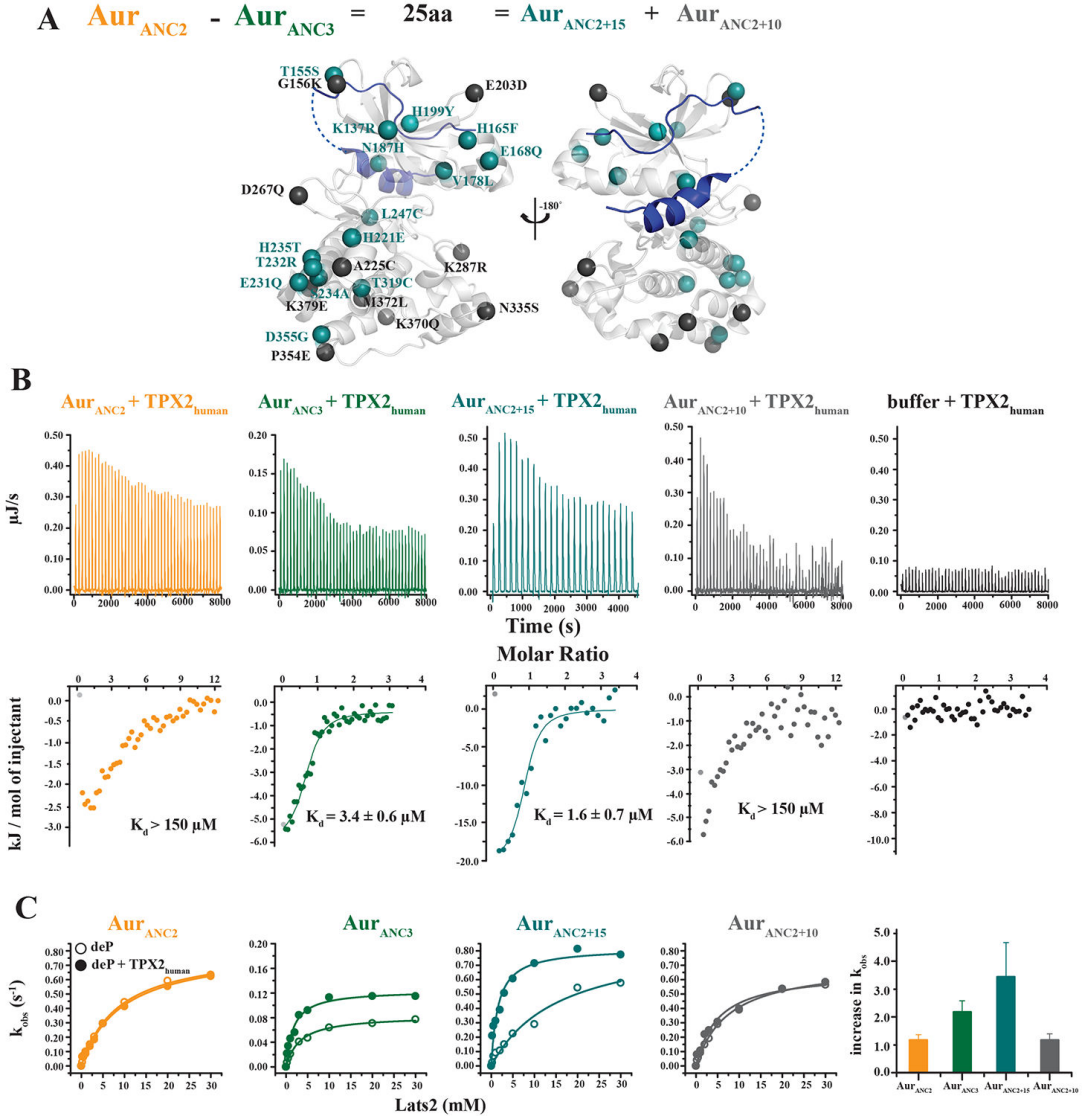
Identification of the allosteric network in Aurora kinase for TPX2 activation. (A) 25 amino acid differences between Aur_ANC2_ that lacks TPX2 allosteric activation and Aur_ANC3_ that has evolved allosteric TPX2 activation are subdivided into constructs Aur_ANC2+15_ and AurANC_2+10_, introducing into Aur_ANC2_ 15 or 10 residues that are either fully conserved or divergent in younger, TPX2-responsive Auroras, respectively. These residues are plotted onto the AurA/TPX2 structure in teal (Aur_ANC2+15_) or grey (Aur_ANC2+10_). (B, C) Tight TPX2 binding as measured by ITC (B), and allosteric activation (C) are both established in Aur_ANC2+15_ but not in the control Aur_ANC2+10_, identifying the 15 residues shown in teal in (A) as sufficient to create allosteric activation by TPX2. Data for Aur_ANC2_ and Aur_ANC3_ are shown for reference. Error bars in (B) represent the standard error for the estimate of K_d_ from the isotherms and are a measure of the goodness of fit of the data. Error bars for increase in k_obs_ in (C) are calculated using jackknifing and error propagation.

If the evolution of Aurora itself is the origin of allosteric control, can the allosteric network in the kinase be identified? Ancestral resurrection provides a tractable approach to this question, since Aur_ANC2_ lacking allosteric activation and Aur_ANC3_ exhibiting allosteric activation differ by only 25 amino acids. Eighteen of those residues are fully conserved in Aur_ANC3_, Aur_ANC4_ and AurA_human_, but not in Aur_ANC1_ and Aur_ANC2_. Three of those 18 residues are not conserved in AurB_human_ (Fig. S10A). Therefore, the 15 remaining fully conserved residues were introduced into Aur_ANC2_ (Aur_ANC2+15_) with the goal of creating allosteric regulation (Fig. 4A). As a control, the non-conserved 10 amino acids were introduced into Aur_ANC2_ (Aur_ANC2+10_). Both engineered kinases are fully active enzymes when phosphorylated on the activation loop T288 (Fig. S10B). Aur_ANC2+15_ showed full restoration of TPX2 binding as well as allosteric activation as seen in Aur_ANC3_ (Fig. 4B, C), suggesting that this network of residues is sufficient to encode for the allosteric response in AurA_human_. Importantly, a further reduction of this network into fewer residues including only the subset located around the R- or C-spine did not result in allosteric response (Fig. S11), buttressing our model of an extended network in Aurora as the origin of allosteric activation by the partner TPX2. Aur_ANC2+10_ lacks both the ability to bind TPX2 and to be activated by it, and is identical to the original Aur_ANC2_ in these properties. We note that these experiments do not show that all 15 residues together are responsible for converting a nonregulated Aurora into an allosterically regulated Aurora, but that at least a subset of this combination of those 9 and 6 residues are essential. This allosteric network for activation by TPX2 is different from that responsible for activation via activation loop T288 phosphorylation, and spans the entire kinase from the TPX2-binding site on top of the N-lobe to the C-lobe (Fig. 4A and Fig. S11A).

Exposing the molecular mechanism of evolving two distinct allosteric regulations in one kinase reveals general evolutionary principles as well as new knowledge about the nature of allosteric networks in the modern kinase. Autophosphorylation of the activation loop as the most ancient activation mechanism can be rationalized because the catalytic machinery for kinase activity was already present during early eukaryotic evolution and was exploited for auto-regulation. Around one billion years ago, with the appearance of the microtubule-associated protein TPX2, Aurora evolved binding to TPX2. This initial binding event may have provided an evolutionary advantage via direct localization of Aurora to the mitotic spindle, and binding remained constant up to modern days. Our data reveal strong co-conservation over long evolutionary periods for the protein/protein interaction (Fig. 3B and Fig. S2). As the last evolutionary event, successive mutations in the kinase gradually dialed in allosteric activation upon TPX2 binding, but with almost no coevolution (Fig. 3), contrary to the notion of reciprocal change for such interactions.

The phenomenon of allosteric coevolution has been of intense interest as it poses a unique challenge to Darwinian evolution (22-25). From a first perspective it would seem that the two proteins making up the system would have to evolve simultaneously in order to function correctly, a challenge referred to as the “irreducibly complex system” problem (22). This would require both the kinase and its modulator to be “pre-set” for allosteric control, a statistically unlikely occurrence. On the other hand, if evolutionary complexity could be reduced by a step-wise evolution starting with colocalization of proteins followed by evolving allostery, this would allow for nonspecific surface residue contacts to evolve into productive interactions (23), followed by mutations only in the kinase domain to evolve allosteric regulation. This perceptive model of colocalization as the first step towards allostery was logically derived in a thoughtful review by Kuriyan and Eisenberg (23).

Colocalization as a catapult towards coevolution in signaling has traditionally been explored in two-component signal transduction systems using directed evolution and computational biology (26, 27). Although very powerful, these techniques lack information on intermediates along the allosteric evolution continuum starting with the primitive ancestral forms, as they investigate only modern sequences (28). More recently, the elegant study by Coyle *et al*. (29) on allosteric regulation of MAP (Mitogen Activated Protein) kinase by its effector Fus3 suggested that more primitive protein MAP kinases have a rugged conformational landscape yielding a diverse set of potential allosteric sites that could be explored by the regulator Fus3. While this proposed evolutionary model is compelling, the study again relied on comparison of only modern-day proteins, therefore not providing direct evidence for evolutionary pathways. Given the generality of autophosphorylation and protein/protein interaction in kinase signaling, resurrecting evolutionary pathways to understand kinase regulation as established here for Aurora A kinase promises to shed light into how differential signaling in the protein kinase superfamily evolved, a question highlighted and discussed in a recent review (30). Moreover, this new approach led to the identification of a subset of residues that created allosteric activation in Aurora, therefore opening a new potential for characterizing atomistic differences between allosteric regulatory networks in the kinase superfamily that can further be exploited in the design of specific allosteric inhibitors or activators.

## Supplementary Material

1
