# Supplementary material for: Ancient Origins of Allosteric Activation in a Ser/Thr Kinase
Source: Science. Author manuscript; Available in PMC 2022 Oct 29. (PMC9617290; doi:10.1126/science.aay9959)
Supplement: 1 [file NIHMS1842691-supplement-1.pdf]

Supplementary Materials for

Ancient Origins of Allosteric Activation in a Ser/Thr Kinase

Adelajda Hadzipasic, Christopher Wilson, Vy Nguyen, Nadja Kern, Chansik Kim, Warintra Pitsawong, Janice Villali, Yuejiao Zheng and Dorothee Kern *

*Corresponding author. Email: dkern@brandeis.edu

This PDF file includes:

Materials and Methods

Figs. S1 to S11

References and notes

Materials and Methods

Ancestral Sequence Reconstruction

Ancestral sequence reconstruction is highly dependent on an accurate phylogeny and multiple sequence alignment. Kinases domain sequences were selected by first performing a BLASTp search against the Uniprot database using an expected value of 1E-15 and Aurora A (human) as a query. The search resulted in 2198 sequences. Bayesian inference of phylogeny is computationally intensive and incapable of handling such a large number of sequences, and we followed the well-described trimming steps for building phylogenetic trees (*1-3*). Focusing on Aurora and its surrounding families (AGC, CAMK and CAMKL) we removed sequences with more than 85% identity, incomplete sequences and sequences with large insertions or deletions. We showed preference for sequences contained within the KinBase database for annotation purposes (*4*). This resulted in a set of 79 sequences spanning the AGC, CAMK, CAMKL, and Aurora families. A robust phylogenetic tree was constructed using the software package BAli-Phy (*5*), which estimates the alignment and the tree simultaneously using Bayesian methodology. The analysis was performed using the RS07 insertion/deletion model, LG amino acid substitution matrix, estimating equilibrium amino acid frequencies, with gamma distributed rates across sites (four categories). Two independent MCMC chains were run until the ASDSF and PSRF-80% CI criteria fell below 0.01 and 1.01, respectively. Eukaryotic Ser/Thr kinases show strong sequence conservation (36.1% identical sites and 48.5% similarity across selected sequences). The strength of phylogeny is illustrated by strong posterior probabilities along branch sites (Fig. S1A).

We choose to resurrect four nodes along the lineage from the divergence of PLK4 and IPL1-Aurora subfamilies of Ser/Thr kinases towards modern day Aurora A in humans. Ancestral sequences were inferred using the marginal likelihood method implemented in PAML (*6*), with

the maximum *a posteriori* phylogeny and expected parameters (normalized equilibrium frequencies and gamma shape parameter) estimated from the BAli-Phy run. The majority sites in the reconstructed sequences have a probability of greater than 0.95 (Fig S1B) as being correctly identified. To account for uncertainty in the reconstruction we generated two additional sequences from key nodes (Aur_{ANC2} and Aur_{ANC3}) by weighted random sampling of the posterior probability distribution generating Aur_{ANC2}^{rep0}, Aur_{ANC2}^{rep1}, Aur_{ANC3}^{rep0} and Aur_{ANC3}^{rep1} (*7*).

While the reconstruction of the kinase domain was relatively straightforward due to its conservation, construction of the TPX2 tree proved to be challenging due to variability in the presence of the three functional TPX2 elements: the Aurora A-, importin- and kinesin-binding domains among the family members. We performed a co-estimated alignment and tree construction as described above for 35 importin and kinesin binding domains spanning plants to animals. While the resulting phylogenetic tree contained high support values, areas of the tree did contain values under 0.5 (Fig. S1D). Using PAML (as described above), we resurrected 2 ancestors, $TPX2_{ANC3}$ and $TPX2_{ANC4}$, that correlated in evolutionary time to Aur_{ANC3} and Aur_{ANC4}. Finally, we used the time tree software to estimate time for ancestral Aurora kinase (*8*) (Fig. S1E).

Cloning and Purification of Aurora A Kinase

Dephosphosphorylated $AurA_{human}$ (residues 122-403, TEV-cleavable, N-terminal His6-tagged) was obtained through a Lambda Protein Phosphatase (LPP) co-expression system. Codon-optimized $AurA_{human}$ in pET28a and untagged LPP in T7-7 plasmid were co-transformed in BL21(DE3) cells and spread on Kan/Amp 2xYT plates. The most robust colony was used for a TB pre-culture and later on to inoculate a 1L culture to an OD = 0.2. Cells were induced with 0.6 mM IPTG for 13-15 hours at 21 °C. Cells were centrifuged at 5000 rpm for 15 mins,

resuspended in Buffer A, and sonicated in the presence of EDTA-free protease inhibitor cocktail and DNAse for 4 mins (20 s on, 20 s off, 3.5 V). Lysates thus obtained were filtered using a 0.22 μm filtering unit and passed through a NiNTA column. The protein was eluted at 100% Buffer B and $AurA_{human}$ fractions were pooled and TEV-cleaved (1 : 20 $AurA_{human}$: TEV molar ratio) and further dephosphorylated with recombinantly purified GST-LPP (20 : 1 $AurA_{human}$: GST-LPP molar ratio) overnight at 4 °C in a 5 kDa dialysis cassette that was exchanged against buffer D. Cleaved $AurA_{human}$ was passed through tandem nickel – GST columns to remove any uncleaved reactants, TEV-protease and GST-LPP, and then purified to homogeneity through a 26/60 S200 size exclusion column. Protein thus produced was aliquoted and flash-frozen before being stored at -80°C and used for biochemical and biophysical work.

Buffer A: 50 mM TrisHCl, 300 mM NaCl, 40 mM imidazole, 20 mM $MgCl_2$, 10 % glycerol, pH = 8.0

Buffer B: 50 mM TrisHCl, 300 mM NaCl, 500 mM imidazole, 20 mM $MgCl_2$, 10 % glycerol, pH = 8.0

Buffer C: 20 mM TrisHCl, 200 mM NaCl, 20 mM $MgCl_2$, 5 mM TCEP, 10 % glycerol, pH = 7.5

Buffer D: 20 mM TrisHCl, 200 mM NaCl, 1 mM $MnCl_2$, 5 mM TCEP, 10 % glycerol, pH = 7.5

Buffer E: 50 mM TrisHCl, 10 mM glutathione, pH = 8.0

TEV-cleavable, N-terminal His6-tagged Aurora ancestors (residues 133-388 in equivalent $AurA_{human}$ numbering) were also cloned and expressed as detailed above. For increased solubility and stability, for all ancestral Aurora proteins, the His6-tag was not cleaved. Purification steps were followed as detailed in (*9*), but skipping the TEV-cleavage step. $AurA_{human}$ did not have a solubility tag as previously discussed (*9*), and controls were performed that showed that cleaved

and uncleaved $AurA_{human}$ behaved the same. Typical yields for phosphorylated / dephosphorylated and T288V Aurora proteins were 8-10 mg / 20-45 mg / 20-25 mg per liter of *E. coli* culture.

$TPX2_{human}$ was subcloned into pET28a and expressed as previously detailed in (*10*). Ancestral TPX2s ($TPX2_{ANC3}$ or $TPX2_{ANC4}$, residues 1 - 45), thrombin-cleavable, His6-tagged, were also subcloned into pET28a and expressed in BL21 (DE3) *E. coli* cells for 3 h at 37 °C. Cells were pelleted, resuspended in Buffer A (50 mM TrisHCl, 300 mM NaCl, 20 mM $MgCl_2$, pH 8.0), sonicated and centrifuged as detailed above, and then passed through a Ni^{2+} column equilibrated in Buffer A. A 10 % Buffer B (50 mM TrisHCl, 300 mM NaCl, 500 mM imidazole, 20 mM $MgCl_2$, pH 8.0) elution step (to remove contaminants), was followed by a 100 % Buffer B elution step (to elute TPX2). TPX2, either modern or ancestral, was further purified to homogeneity through a 26/60 S75 size exclusion column equilibrated in Buffer C (20 mM TrisHCl, 20 0mM NaCl, 20 mM $MgCl_2$, 5 mM TCEP, 10 % glycerol, pH 7.50). TPX2 thus produced was aliquoted and flash-frozen before being stored at -80 °C and used for biochemical and biophysical studies. Typical yields were 50 - 60 mg of TPX2 per liter of *E. coli* culture. Uncleaved TPX2s were found to have better long-term stability, therefore the tags were left on (after control experiments that showed the same results in activity and binding experiments).

***In Vitro* Kinase Assays**

A. ADP/NADH Coupled Assay

Kinetic parameters for the kinases, such as K_M^{ATP}, K_M^{MgCl2}, K_M^{Lats2}, k_{obs} in the presence or absence of $TPX2_{human}$, $TPX2_{ANC3}$ and $TPX2_{ANC4}$, were assessed through the ADP/NADH coupled assay which relates peptide phosphorylation (as measured through ADP production) to the oxidation of NADH to NAD^+ (*10, 11*). A 96-well microplate reader was used (SpectraMax) and

assays were performed in a white, clear-bottom, half-well 96-well plates (Corning #3994) in a final reaction volume of 100 µL per well.

Assays were performed at 25 °C in assay buffer (20 mM TrisHCl, 200 mM NaCl, 20 mM $MgCl_2$, 5 mM TCEP, 10% glycerol, pH 7.5) supplemented with 6.6 mM phosphoenolpyruvate (PEP), 725 µM NADH, 370 nM pyruvate kinase (PK), 350 nM lactate dehydrogenase (LDH), and 0.6 mg/mL BSA. Oxidation of NADH was monitored at A_{340} for 20 mins using SOFTmaxPRO software. Appropriate controls (ensuring PEP/NADH/PK/LDH are not limiting factors) were carried out before data was gathered.

Lats2 peptide (ATLARRD**S**LQKPGLE) and INCENP peptide (HPPNLLELFGTILPLDLEDIFKKS) were obtained through Genscript. Aurora A phosphorylates Lats2, a physiological substrate of the kinase, on a Ser residue (bolded and underlined, above). K_M^{Lats2} and k_{obs}^{Lats2} were initially measured for 1 µM Aurora A in the presence of increasing amounts of peptide (0, 0.2, 0.5, 1, 2, 3, 5, 10, 20 and 30 mM Lats2). In experiments with activating TPX2, less Aurora A was used to avoid reaction completion during the dead time of the instruments (0.25 µM Aurora A and 50 µM TPX2). The other Aurora concentrations used are shown for each figure legend.

B. Reverse Phase HPLC Assay

Modern and ancestral Aurora kinases were mixed with 1mM Kemptide (LRRA**S**LG, synthesized by Genscript) in the absence or presence of TPX2 in kinase buffer (20 mM TrisHCl, 200 mM NaCl, 3% (v/v) glycerol, 20 mM $MgCl_2$, 5 mM TCEP, pH 7.5) at 25 °C. Ser residue that Aurora phosphorylates is shown in bold and underlined. TPX2 concentrations varied depending

on the experiment. Please refer to the figure legends for more detail. Assay was run as detailed in (*9*).

Isothermal Titration Calorimetry (ITC)

Aurora and TPX2, modern and ancestral proteins, were dialyzed overnight 4 °C in ITC buffer (20 mM TrisHCl, 200 mM NaCl, 10% (v/v) glycerol, 1 mM TCEP, pH 7.5), in preparation for ITC runs. For tight Aurora: TPX2 interactions, Aurora: TPX2 protein ratios were typically 1:7 or 1:10. Specifically, 30-40 μM or 50-60 μM Aur_{ANC3} and alternates (Aur_{ANC3}^{rep0} / Aur_{ANC3}^{rep1}), Aur_{ANC4}, $AurA_{human}$ and $Aur_{ANC2+15}$ were tested for binding to 210-280 μM or 300-400 μM or 500-600 μM $TPX2_{ANC3,}$ $TPX2_{ANC4}$ or $TPX2_{human}$. For weak Aurora: TPX2 interactions, Aurora: TPX2 protein ratios were typically 1:30 or 1:40. Specifically, 30-40 μM Aur_{ANC1}, Aur_{ANC2} and alternates (Aur_{ANC2}^{rep0} / Aur_{ANC2}^{rep1}), and $Aur_{ANC2+15}$ were tested for binding to 900-1200 μM or 1200-1600 μM $TPX2_{ANC3,}$ $TPX2_{ANC4}$ or $TPX2_{human}$.

Experiments were run on a Nano ITC (TA instruments), with a 170 μL cell volume and 50 μL titrant volume, added in 1 μL increments, every 180 s while stirring at 350 rpm and at 25 °C. In experiments with low heat rate observed, titrants were added in 2 uL increments. The isotherms were analyzed via the NanoAnalyze Software using the Independent Fit model.

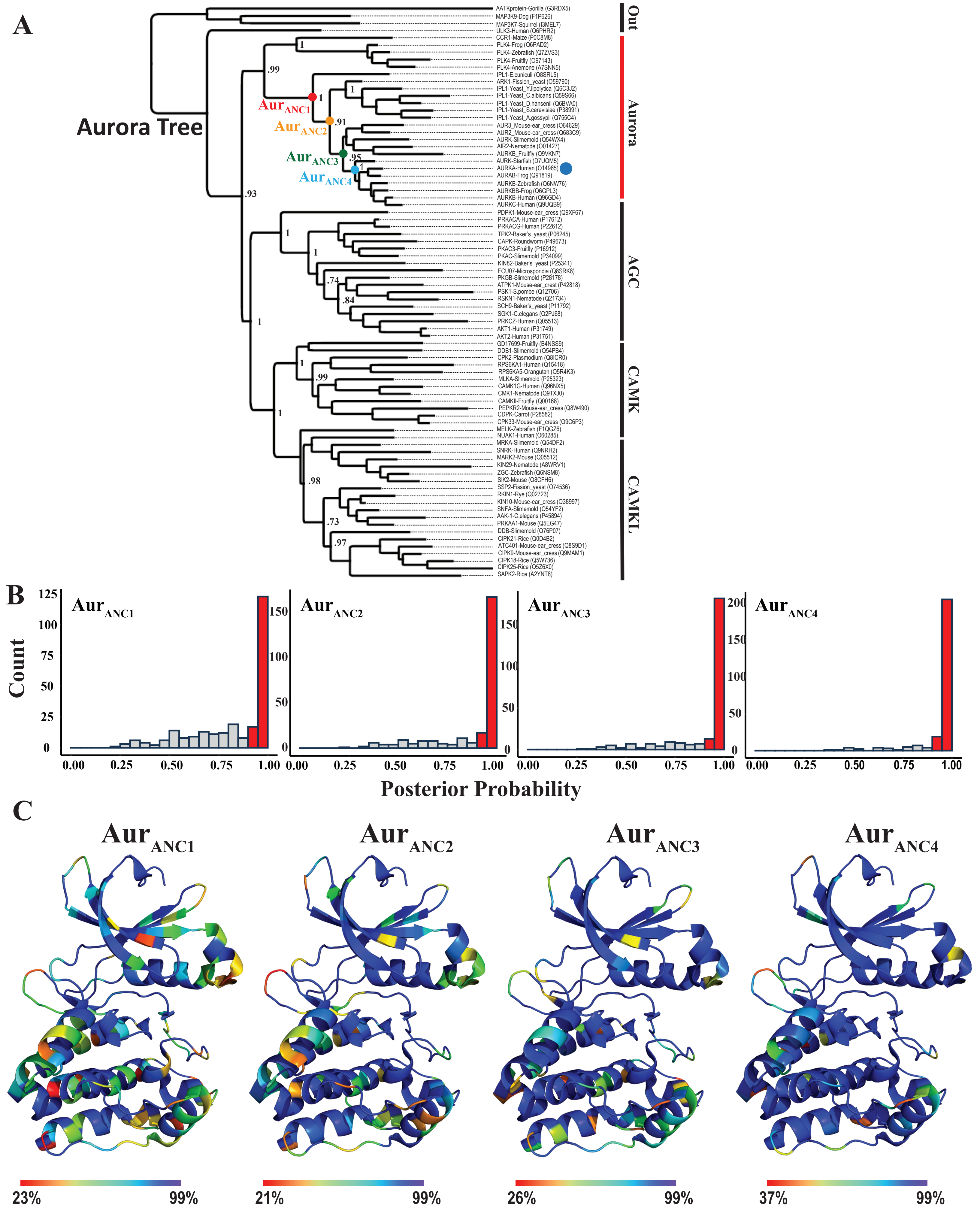

A
Aurora Tree
AATKprotein-Gorilla (G3RDX5)
MAP3K9-Dog (F1P626)
MAP3K7-Squirrel (I3MEL7)
ULK3-Human (Q6PHR2)
CCR1-Maize (P0C8M8)
PLK4-Frog (Q6PAD2)
PLK4-Zebrafish (Q7ZVS3)
PLK4-Fruitfly (O97143)
PLK4-Anemone (A7SNN5)
IPL1-E.cuniculi (Q8SRL5)
ARK1-Fission_yeast (O59790)
IPL1-Yeast_Y.lipolytica (Q6C3J2)
IPL1-Yeast_C.albicans (Q59S66)
IPL1-Yeast_D.hansenii (Q6BVA0)
IPL1-Yeast_S.cerevisiae (P38991)
IPL1-Yeast_A.gossypii (Q755C4)
AUR3_Mouse-ear_cress (O64629)
AUR2_Mouse-ear_cress (Q683C9)
AURK-Slimemold (Q54WX4)
AIR2-Nematode (O01427)
AURKB_Fruitfly (Q9VKN7)
AURK-Starfish (D7UQM5)
AURKA-Human (O14965)
AURAB-Frog (Q91819)
AURKB-Zebrafish (Q6NW76)
AURKBB-Frog (Q6GPL3)
AURKB-Human (Q96GD4)
AURKC-Human (Q9UQB9)
PDPK1-Mouse-ear_cress (Q9XF67)
PRKACA-Human (P17612)
PRKACG-Human (P22612)
TPK2-Baker's_yeast (P06245)
CAPK-Roundworm (P49673)
PKAC3-Fruitfly (P16912)
PKAC-Slimemold (P34099)
KIN82-Baker's_yeast (P25341)
ECU07-Microsporidia (Q8SRK8)
PKGB-Slimemold (P28178)
ATPK1-Mouse-ear_crest (P42818)
PSK1-S.pombe (Q12706)
RSKN1-Nematode (Q21734)
SCH9-Baker's_yeast (P11792)
SGK1-C.elegans (Q2PJ68)
PRKCZ-Human (Q05513)
AKT1-Human (P31749)
AKT2-Human (P31751)
GD17699-Fruitfly (B4NSS9)
DDB1-Slimemold (Q54PB4)
CPK2-Plasmodium (Q8ICR0)
RPS6KA1-Human (Q15418)
RPS6KA5-Orangutan (Q5R4K3)
MLKA-Slimemold (P25323)
CAMK1G-Human (Q96NX5)
CMK1-Nematode (Q9TXJ0)
CAMKII-Fruitfly (Q00168)
PEPKR2-Mouse-ear_cress (Q8W490)
CDPK-Carrot (P28582)
CPK33-Mouse-ear_cress (Q9C6P3)
MELK-Zebrafish (F1QGZ6)
NUAK1-Human (O60285)
MRKA-Slimemold (Q54DF2)
SNRK-Human (Q9NRH2)
MARK2-Mouse (Q05512)
KIN29-Nematode (A8WRV1)
ZGC-Zebrafish (Q6NSM8)
SIK2-Mouse (Q8CFH6)
SSP2-Fission_yeast (O74536)
RKIN1-Rye (Q02723)
KIN10-Mouse-ear_cress (Q38997)
SNFA-Slimemold (Q54YF2)
AAK-1-C.elegans (P45894)
PRKAA1-Mouse (Q5EG47)
DDB-Slimemold (Q76P07)
CIPK21-Rice (Q0D4B2)
ATC401-Mouse-ear_cress (Q8S9D1)
CIPK9-Mouse-ear_cress (Q9MAM1)
CIPK18-Rice (Q5W736)
CIPK25-Rice (Q5Z6X0)
SAPK2-Rice (A2YNT8)
Out
Aurora
AGC
CAMK
CAMKL
Aur ANC1
Aur ANC2
Aur ANC3
Aur ANC4
1
.99
.91
.95
.93
.74
.84
.98
.73
.97
B
Count
Posterior Probability
0.00
0.25
0.50
0.75
1.00
125
100
75
50
25
0
150
200
C
23%
21%
26%
37%
99%

D

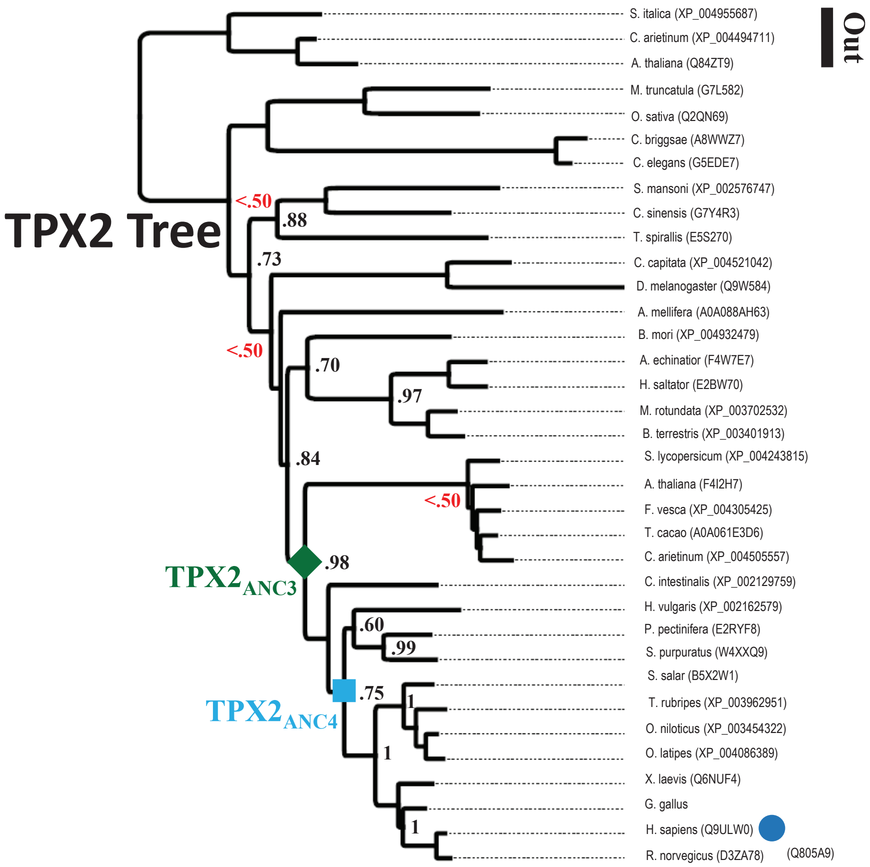

E

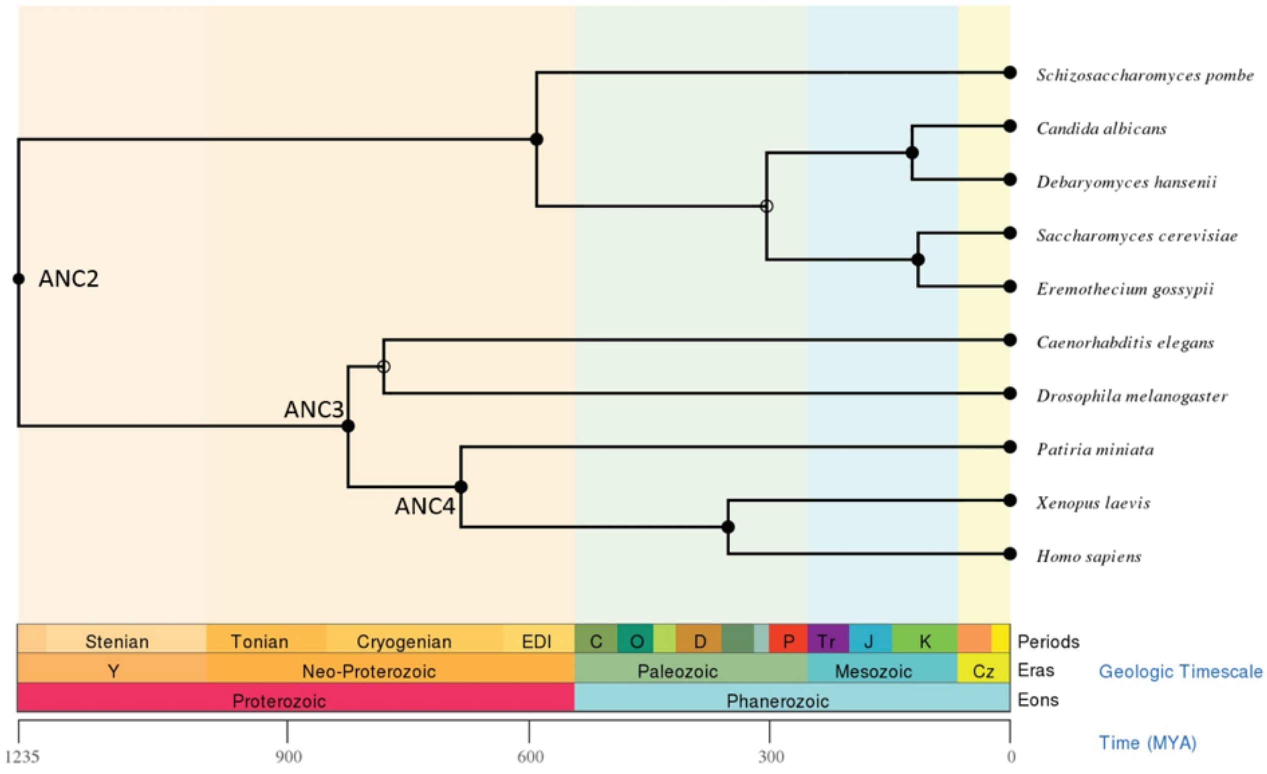

Fig. S1. Aurora and TPX2 trees. (A) Phylogenetic tree of Aurora and closely related Ser/Thr kinases calculated with BAli-Phy (*5*) with posterior probabilities of the nodes shown and the UniProt/UniParc accession codes shown in brackets. (B) Histogram of the posterior probabilities; >0.9 colored red and <0.9 are colored gray. (C) Mapping of the posterior probabilities onto the AurA structure. (D) Phylogentic tree of TPX2 using the AurA-binding sequence of 1-45. (E) Estimated times for ancestral Aurora kinases calculated using the time tree software (*8*). Periods, eras and eons, along with a timeline, are displayed at the bottom.

A

```
                    140        150        160        170        180        190        200        210        220
Aur ANC1    FEIGKLLG KGKFGRVYLA REKETGFIVA LKVIHKKELQ KAKVEHQVRR EIEIHSNLRH PNILRLYGHF HDEDNVYLIL EYAARGELYK
Aur ANC2    FEIGKPLG KGKFGRVYLA REKKTGFIVA LKVLHKSELQ KAKVEHQVRR EIEIQSNLRH PNILRLYGHF HDEKRVYLIL EYAARGELYK
Aur ANC3    FEIGRPLG KGKFGRVYLA REKKSKFIVA LKVLFKSQLQ KAKVEHQLRR EIEIQSHLRH PNILRLYGYF HDDKRVYLIL EYAARGELYK
Aur ANC4    FEIGRPLG KGKFGNVYLA REKKSKFIVA LKVLFKSQLQ KAKVEHQLRR EIEIQSHLRH PNILRLYGYF HDDTRVYLIL EYAARGELYK
AurA human  FEIGRPLG KGKFGNVYLA REKQSKFILA LKVLFKAQLE KAGVEHQLRR EVEIQSHLRH PNILRLYGYF HDATRVYLIL EYAPLGTVYR

                   230        240        250        260        270        280        290        300        310
Aur ANC1    YLQKAGRFSE EEASHYIRQI ANALSYLHSR HVIHRDIKPE NLLLGSDGEL KIADFGWAVH APSNRRKTMC GTLDYLAPEM VEGKEHDEKV
Aur ANC2    HLQKAGRFSE ETASHYIYQI ANALSYLHSK HVIHRDIKPE NLLLGSDGEL KIADFGWSVH APSNRRKTMC GTLDYLPPEM VEGKEHDEKV
Aur ANC3    ELQKCGRFSE QRAATYIYQI ANALSYCHSK HVIHRDIKPE NLLLGSQGEL KIADFGWSVH APSNRRRTMC GTLDYLPPEM VEGKEHDEKV
Aur ANC4    ELQKCGRFDE QRSATYIYQL ANALSYCHSK KVIHRDIKPE NLLLGSKGEL KIADFGWSVH APSSRRRTMC GTLDYLPPEM IEGKTHDEKV
AurA human  ELQKLSKFDE QRTATYITEL ANALSYCHSK RVIHRDIKPE NLLLGSAGEL KIADFGWSVH APSSRRTTLC GTLDYLPPEM IEGRMHDEKV

                   320        330        340        350        360        370        380      388
Aur ANC1    DLWSLGILTY EFLVGKPPFE SENHNETYKK IKSVDYTFPS HVSPDARDLI SRLLVHDPSK RMSLEEVLNH PWIVKNSQ
Aur ANC2    DLWSLGILTY EFLVGKPPFE SESHNETYKR IKKVDFTFPS HVSPDARDLI SRLLVHDPSK RMSLEEVMKH PWIVKNSQ
Aur ANC3    DLWSLGILCY EFLVGKPPFE SESHSETYKR IKKVDFTFPS HVSEGARDLI SRLLVHDPSQ RLSLEEVMEH PWIVKNSQ
Aur ANC4    DLWSLGVLCY EFLVGKPPFE SESHSETYRR ITKVDFTFPP HVSEGARDLI SKLLKHNPSQ RLSLEGVMEH PWIVKNSQ
AurA human  DLWSLGVLCY EFLVGKPPFE ANTYQETYKR ISRVEFTFPD FVTEGARDLI SRLLKHNPSQ RPMLREVLEH PWITANSS
```

B

```
                   10         20         30         40    45
TPX2 ANC3   MNYFDELYEF SAPKPFVDFT RGPDEGDTDN ADKWFDKAHE YE
TPX2 ANC4   MNYFDERYEF DAPKPFVDFS AGHDEGDTDN ADKWFDKAHE NE
TPX2 human  MSQVKSSYSY DAPSDFINFS SLDDEGDTQN IDSWFEEKAN LENKL
```

Fig. S2. Multiple sequence alignment of human Aurora A and reconstructed ancestral Aurora kinases (A) as well as human TPX2 and its ancestors (B). Non-conserved residues are shown bolded and underlined.

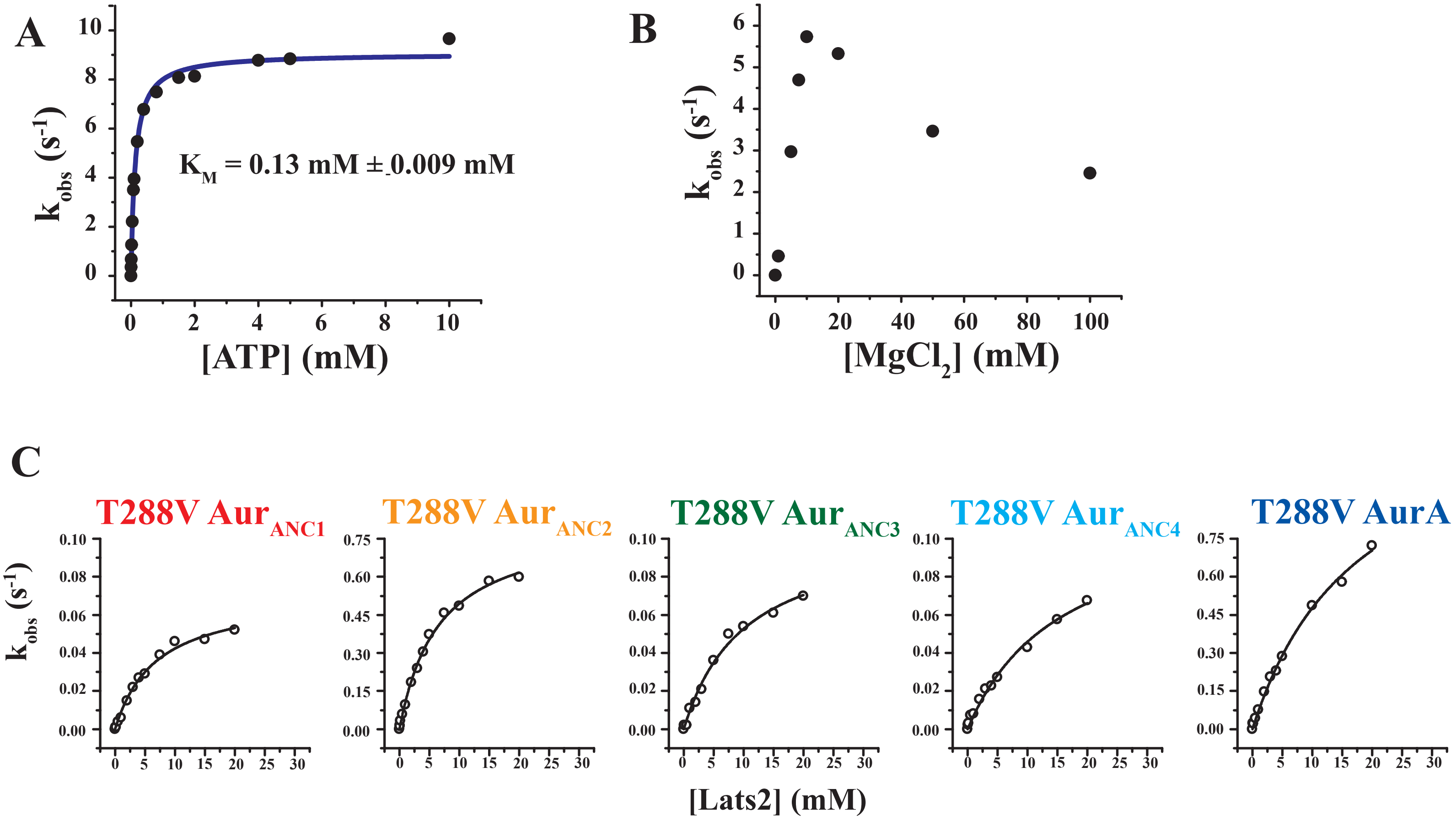

Fig. S3. Enzymatic controls. Dependence of Lats2 phosphorylations rates on ATP (A) and $MgCl_2$ (B) concentrations for 0.05 μM phosphorylated $AurA_{human}$. Based on these data, all kinetic experiments presented in this manuscript were conducted at saturation concentrations for ATP and Mg^{2+} by using 5 mM ATP and 20 mM $MgCl_2$. (C) Enzyme assays of T288V Aurora constructs to mimic the dephosphorylated enzymes were performed under the same experimental conditions as WT dephosphorylated Aurora proteins of Fig. 2. The close similarity of these data verify that the dephosphorylated WT proteins do not undergo autophosphorylation during the enzymatic assays of Lats2 phosphorylation.

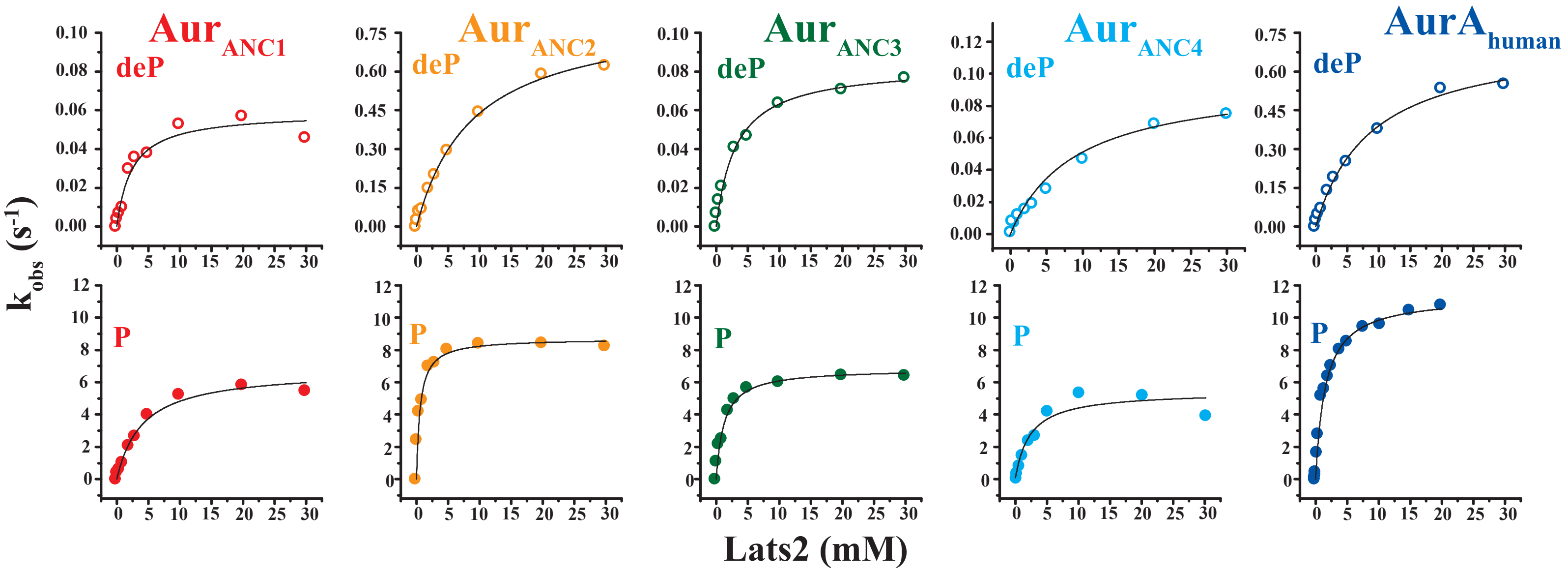

Fig. S4. Michaelis-Menten plots for ancestral and human Aurora with the substrate peptide Lats2 comprising the phosphorylation region of Lats2. Phosphorylation of different concentrations of Lats2 peptide was monitored using the ADP/NADH coupled assay with 1 µM dephosphorylated or 0.05 µM phosphorylated Aurora and 5 mM ATP in assay buffer (20 mM TrisHCl, 200 mM NaCl, 20 mM $MgCl_2$, 10% (v/v) glycerol, 5 mM TCEP, pH 7.50) at 25 °C. Error bars represent the standard error for the estimate of k_{cat} or K_M through the Michaelis-Menten equation and are a measure of the goodness of fit of the data. Error bars for increase in k_{obs} are calculated using jackknifing and error propagation.

A

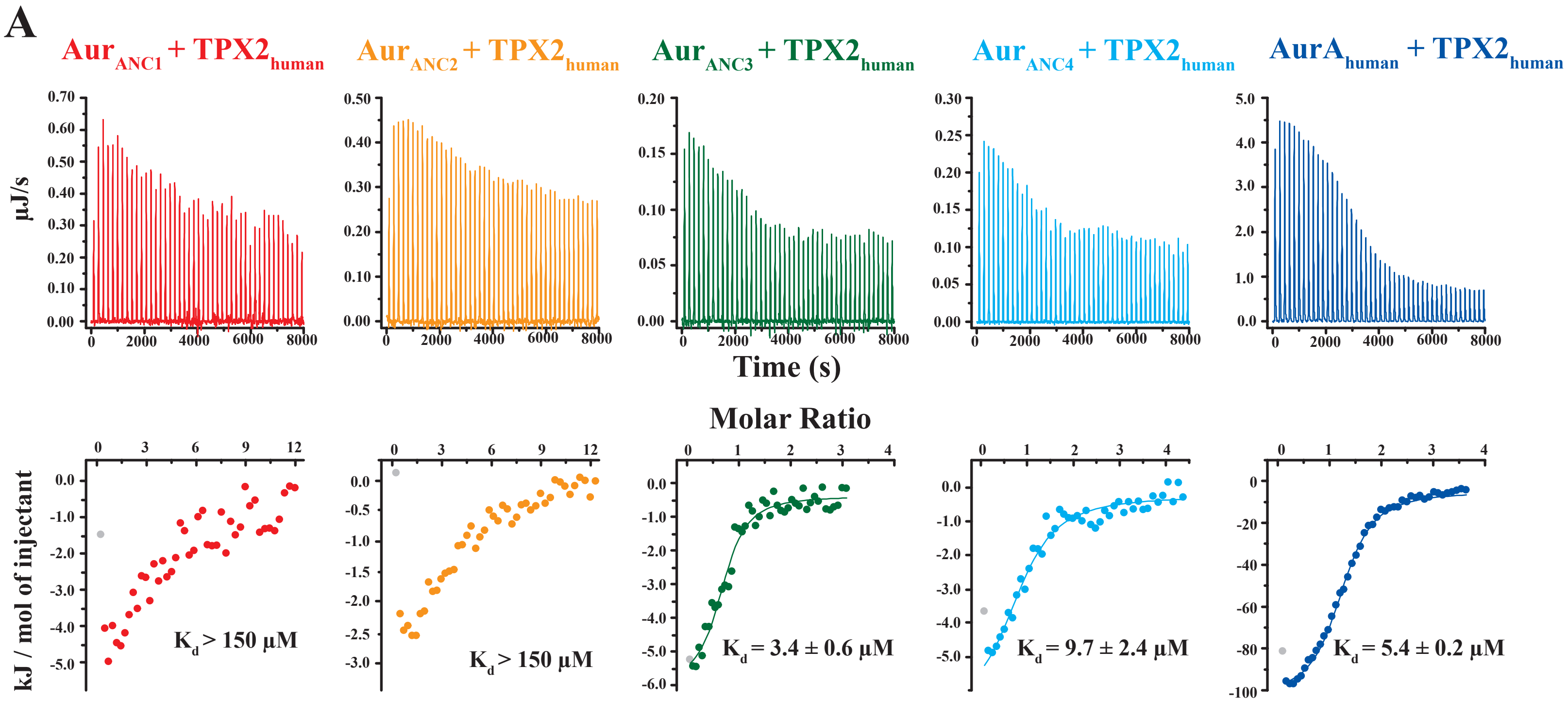

B

	$TPX2_{ANC3}$ Kd (μM)	±	$TPX2_{ANC4}$ Kd (μM)	±	$TPX2_{human}$ Kd (μM)	±
Aur_{ANC1}	>150		>150		>150	
Aur_{ANC2}	>150		>150		>150	
Aur_{ANC3}	7.0	4.5	5.4	1.6	3.4	0.6
Aur_{ANC4}	4.1	1.7	5.8	1.8	9.7	2.4
$AurA_{human}$	6.7	4.3	21.3	8.0	5.4	0.2

Fig. S5. ITC data for $TPX2_{human}$ binding to modern and ancestral Aurora kinases. Representative isotherms (A) and a summary of the binding constants for various Aurora / TPX2 pairs (B).

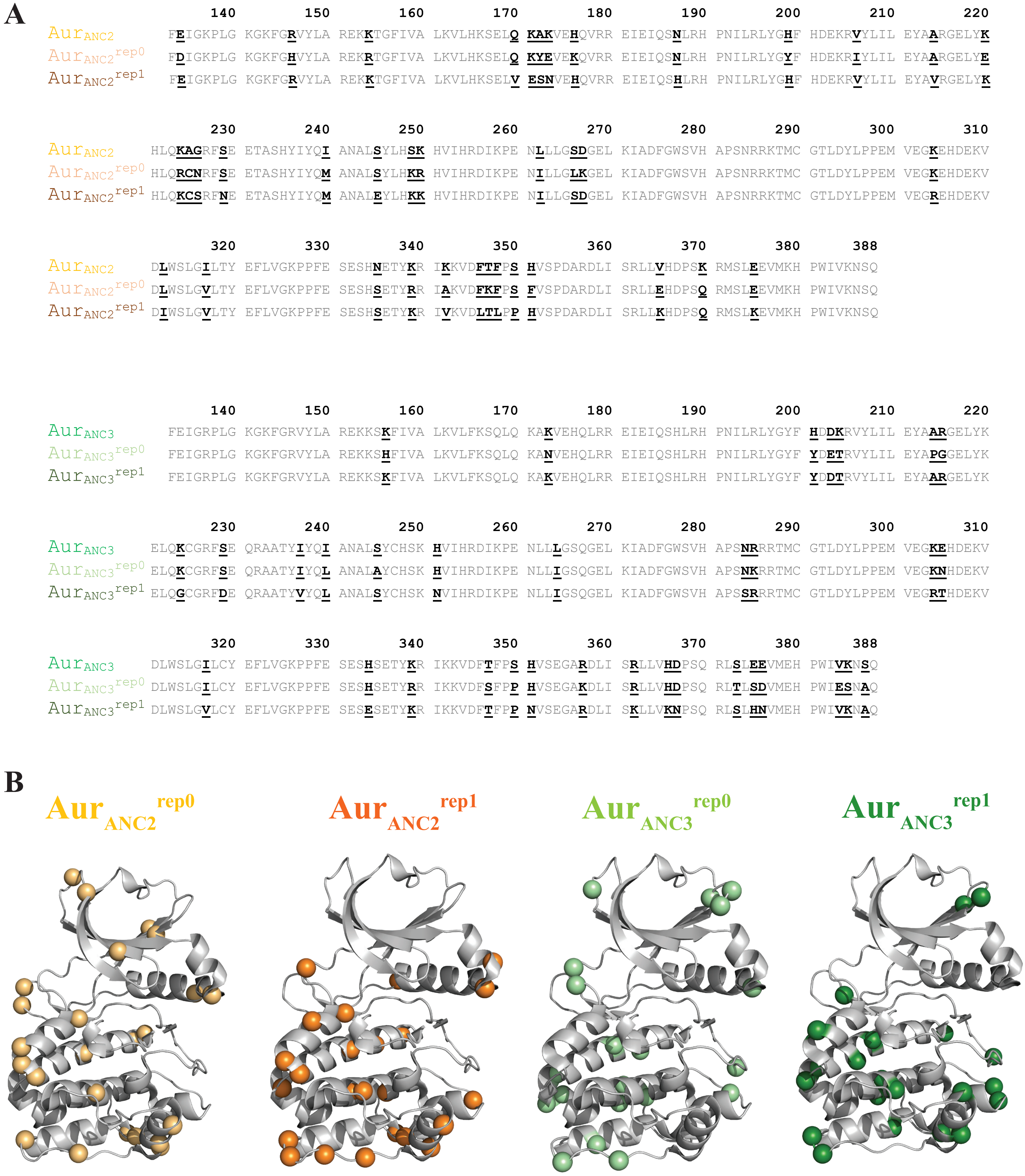

Fig. S6. Alternates from the ancestral posterior probability distribution for the key nodes Aur$_{ANC2}$ and Aur$_{ANC3}$ that were experimentally studied to test the robustness of the ancestral reconstructions. (A) Sequence alignments with altered residues shown bolded and underlined. (B) Amino acid changes in these alternate proteins are plotted onto the AurA structure.

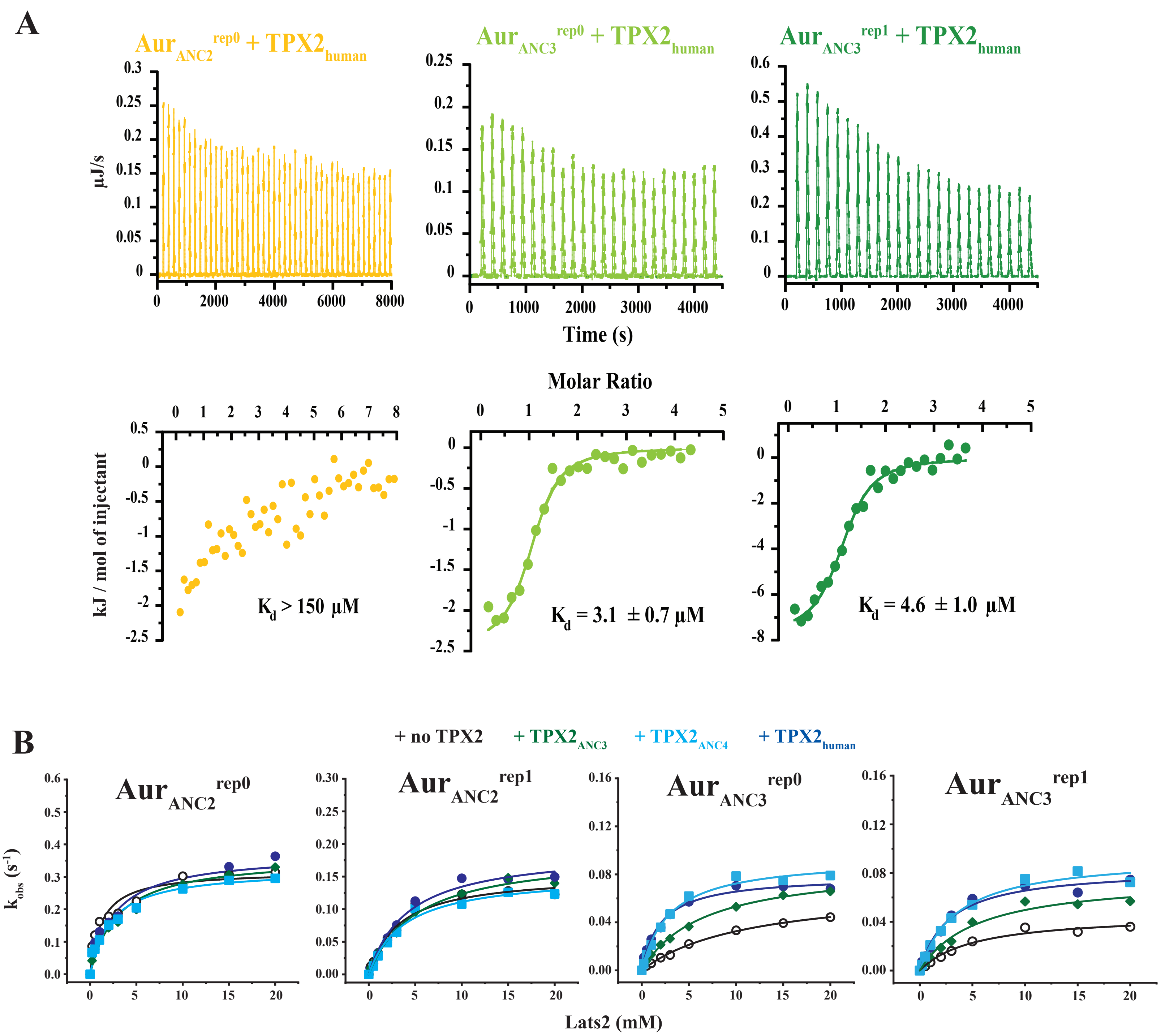

Fig. S7. TPX2 binding and allosteric activation for alternates of Aur_{ANC2} and Aur_{ANC3} to test robustnes of ASR. (A) Dissociation constants (K_d) of $TPX2_{human}$ binding to alternates of Aur_{ANC2} and Aur_{ANC3} sampled from the ancestral posterior probability distribution, measured by ITC (see Fig. S6 for alternate sequences). Binding to Aur_{ANC2}^{rep1} was too weak to be detected. (B) Michaelis Menten plots of alternate Aurora ancestors in the absence and presence of $TPX2_{ANC3}$, $TPX2_{ANC4}$ or $TPX2_{human}$ under the same conditions as Fig. 3C. Specifically, 100µM TPX2 (for 2.5µM Aur_{ANC3}^{rep0} or 2.5µM Aur_{ANC3}^{rep1} assuring saturation) and 500µM TPX2 (for 1µM Aur_{ANC2}^{rep0} or 1µM Aur_{ANC2}^{rep1}) were used. Error bars in (A) represent the standard error for the estimate of K_d from the isotherms and are a measure of the goodness of fit of the data.

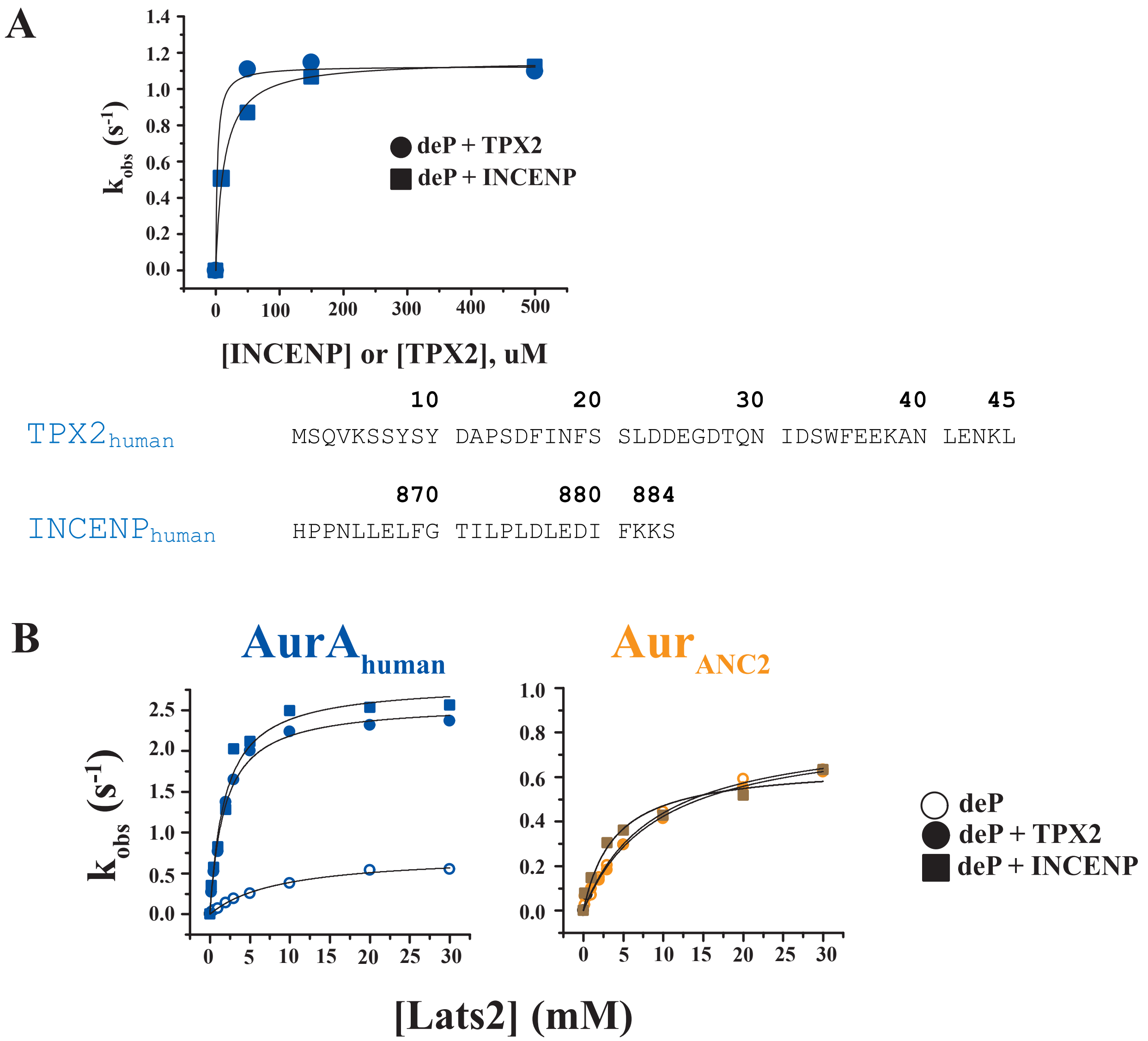

Fig. S8. Specificity for TPX2 versus INCENP (a binding partner of $AurB_{human}$) for Aurora A is encoded in affinity, not in the allosteric activation. (A) Lats2 phosphorylation (3 mM Lats2) measured at 0.25 µM $AurA_{human}$ in the presence of increasing amounts of $TPX2_{human}$ and INCENP. $AurA_{human}$ shows greater affinity for its natural binding partner TPX2 (fitted $K_{act} = 2 \pm 0.2$ µM), than for INCENP (fitted $K_{act} = 12 \pm 4$ µM). Sequences are shown below. (B) $AurA_{human}$ is allosterically activated by TPX2 and INCENP to the same extent when saturated, whereas Aur_{ANC2} is not allosterically activated by either binding partner. 150 µM and 500 µM INCENP were used for $AurA_{human}$ and Aur_{ANC2} assays, respectively.

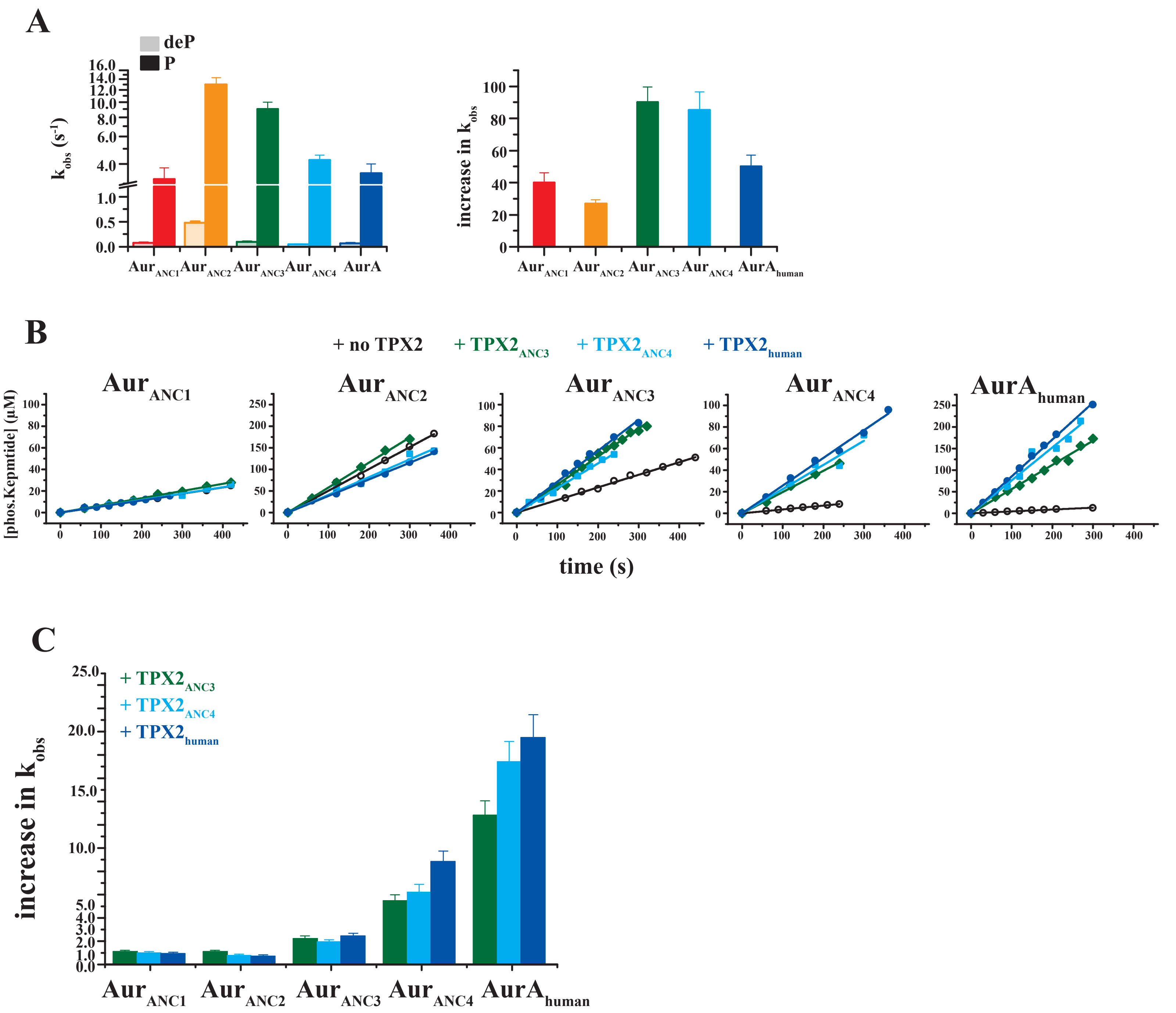

Fig. S9. Evolution of allosteric activation by phosphorylation and TPX2 binding to ancestral and modern Auroras measured using a different substrate (Kemptide) to address the question of possible substrate coevolution. (A) Effect of Aurora's phosphorylation state on k_{obs} using 1 mM Kemptide (absolute value – left, and fold increase in k_{obs} – right). (B) Kemptide phosphorylation (1 mM Kemptide used in the assay) measured for dephosphorylated Aurora kinases in the absence and presence of $TPX2_{ANC3/ANC4/human}$. (C) Fold increase in k_{obs} for 1 mM Kemptide extracted from the data in (B). Kemptide phosphorylation over time was monitored using a discontinuous RP-HPLC assay measuring the decrease in dephosphorylated peptide and increase in phosphorylated peptide over time. Results are very similar to data for phosphorylation of Lats2 (Fig. 3D). Error bars in (A) represent the standard error for the estimate of k_{cat} or K_M through the Michaelis-Menten equation and are a measure of the goodness of fit of the data. Error bars for increase in k_{obs} in (C) are calculated using jackknifing and error propagation.

A

Selection strategy for generating $Aur_{ANC2+15}$ and $Aur_{ANC2+10}$

AA#	137	155	156	165	168	178	187	199	203	221	225	231	232	234	235	247	267	287	319	335	354	355	370	372	379
Aur_{ANC1}	K	T	G	H	E	V	N	H	E	Y	A	E	E	S	H	L	D	K	T	N	P	D	K	M	N
Aur_{ANC2}	K	T	G	H	E	V	N	H	E	H	A	E	T	S	H	L	D	K	T	N	P	D	K	M	K
Aur_{ANC3}	R	S	K	F	Q	L	H	Y	D	E	C	Q	R	A	T	C	Q	R	C	S	E	G	Q	L	E
Aur_{ANC4}	R	S	K	F	Q	L	H	Y	D	E	C	Q	R	A	T	C	K	R	C	S	E	G	Q	L	E
$AurA_{human}$	R	S	K	F	Q	L	H	Y	A	E	L	Q	R	A	T	C	A	T	C	Q	E	G	Q	P	E
$AurB_{human}$	R	S	H	F	Q	L	H	Y	R	E	S	Q	R	A	T	C	K	K	C	N	T	G	E	L	A

B

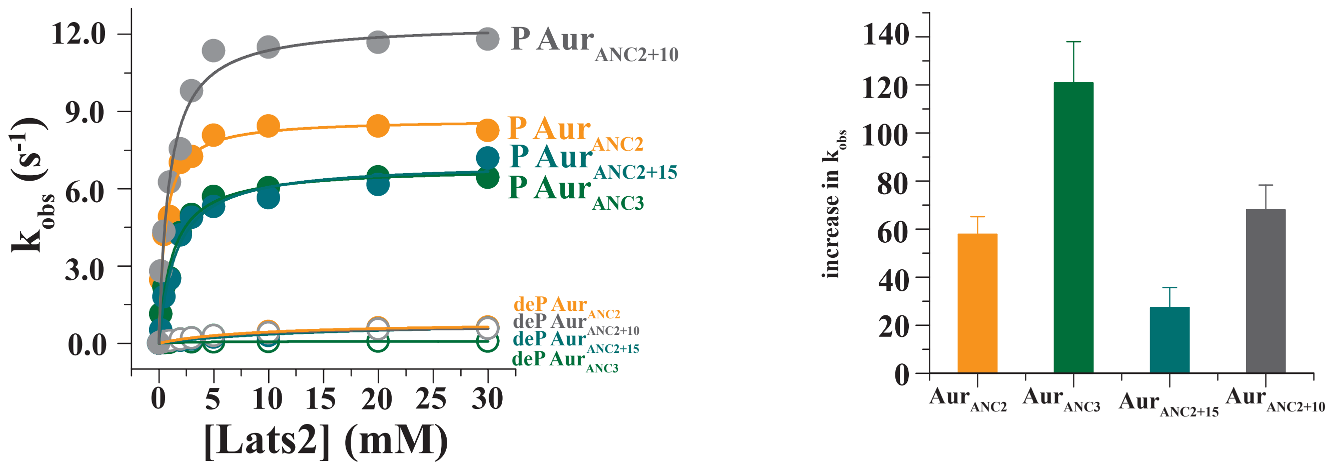

Fig. S10. Strategy for discerning the allosteric network in Aurora (see also Fig. 4). (A) Amino acid differences between ancestral Auroras, $AurA_{human}$ and $AurB_{human}$ are shown. Marked in blue are the 15 residues that change between Aur_{ANC2} (that cannot be allosterically activated by TPX2) and Aur_{ANC3} (that displays allosteric TPX2 activation), and that then stay constant in further evolution from Aur_{ANC3} to $AurA_{human}$ and $AurB_{human}$. Marked in black are the 10 other residues that change between Aur_{ANC2} and Aur_{ANC3} but diverge in evolution either among Aur_{ANC3}, Aur_{ANC4} and $AurA_{human}$ or only in comparison to $AurB_{human}$ (underlined). (B) The resulting $Aur_{ANC2+15}$ and $Aur_{ANC2+10}$ constructs have comparable activities to both dephosphorylated and phosphorylated forms of Aur_{ANC2} verifying that the 15 identified amino acids are solely responsible for creating an allosteric network for TPX2 activation (Fig. 4), but do not alter other enzymatic properties including activation via T288 phosphorylation. Increase in catalysis of 1 mM Lats2 as a result of T288 phosphorylation is shown on the right. Error bars for increase in k_{obs} (B, right) are calculated using jackknifing and error propagation.

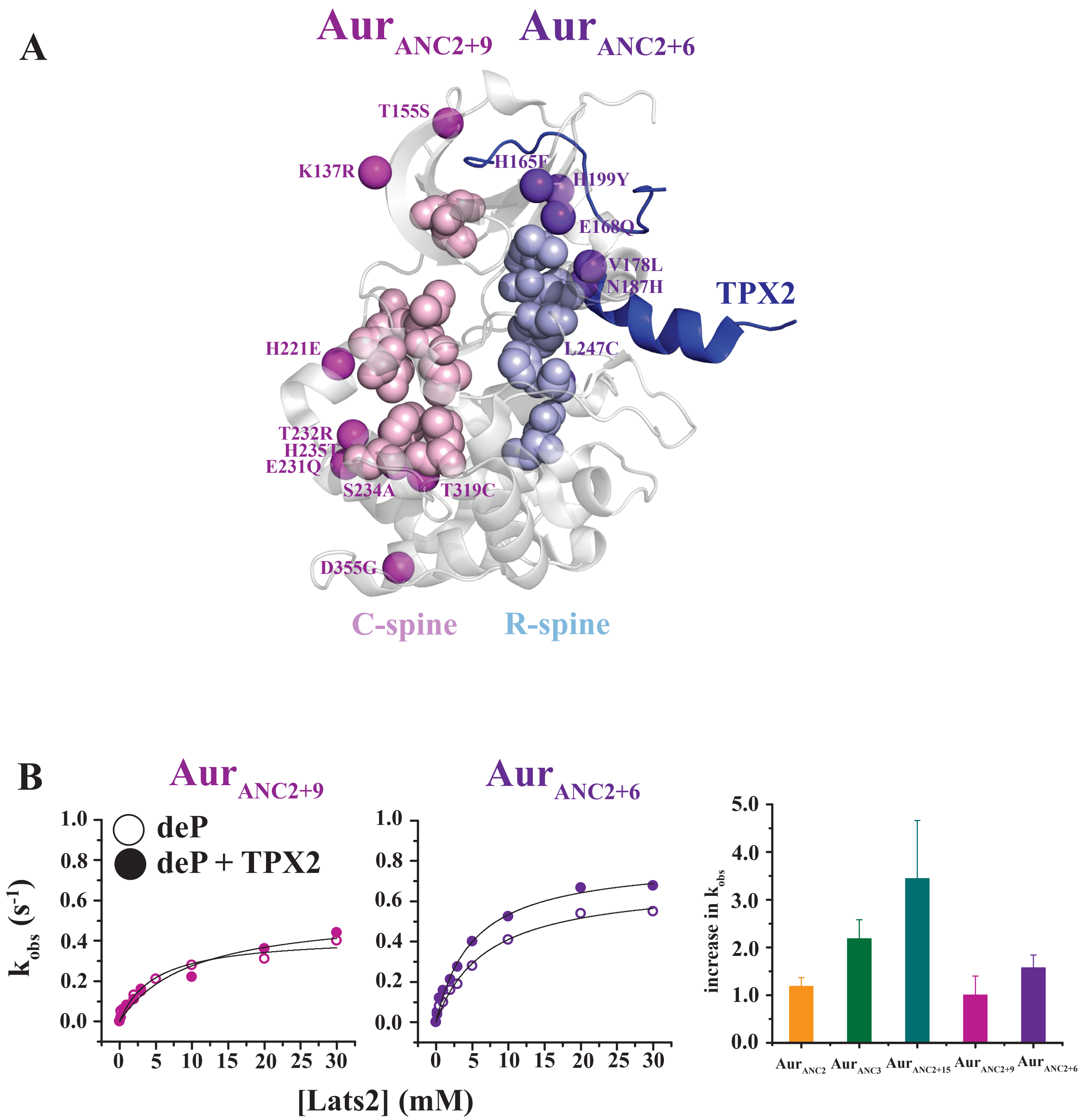

Fig. S11. Subset of the 15 amino acids cannot propagate allosteric activation by TPX2. (A) $Aur_{ANC2+15}$ is subdivided into Aur_{ANC2+9} and Aur_{ANC2+6} to mutate 9 residues that contact the C-spine (shown as van der Waal's representation in pink) or 6 residue that contact the R-spine (shown as van der Waal's representation in light blue) of $AurA_{human}$. Italicized letters represent residues that are in the back of the protein and cannot be seen in the current representation. (B) These two subsets do not recapitulate the effect of allosteric activation of $Aur_{ANC2+15}$ by $TPX2_{human}$. Error bars for increase in k_{obs} (B, right) are calculated using jackknifing and error propagation.

References and notes:

1. J. W. Thornton, E. Need, D. Crews, Resurrecting the ancestral steroid receptor: Ancient origin of estrogen signaling. *Science* **301**, 1714-1717 (2003).
2. V. Hanson-Smith, B. Kolaczkowski, J. W. Thornton, Robustness of ancestral sequence reconstruction to phylogenetic uncertainty. *Molecular biology and evolution* **27**, 1988-1999 (2010).
3. J. I. Boucher, J. R. Jacobowitz, B. C. Beckett, S. Classen, D. L. Theobald, An atomic-resolution view of neofunctionalization in the evolution of apicomplexan lactate dehydrogenases. *Elife* **3**, (2014).
4. G. Manning., (2002). KinBase: Genomics, evolution and function of protein kinases. Retrieved from: http://kinase.com/web/current/kinbase/.
5. B. D. Redelings, M. A. Suchard, Joint Bayesian estimation of alignment and phylogeny. *Syst Biol* **54**, 401-418 (2005).
6. Z. Yang, PAML 4: phylogenetic analysis by maximum likelihood. *Mol Biol Evol* **24**, 1586-1591 (2007).
7. D. D. Pollock, B. S. W. Chang, in *Ancestral Sequence Reconstruction*, D. A. Liberles, Ed. (Oxford University Press, Oxford, 2007).
8. S. B. Hedges, J. Marin, M. Suleski, M. Paymer, S. Kumar, Tree of life reveals clock-like speciation and diversification. *Mol Biol Evol* **32**, 835-845 (2015).
9. A. Zorba *et al.*, Molecular mechanism of Aurora A kinase autophosphorylation and its allosteric activation by TPX2. *Elife* **3**, e02667 (2014).
10. A. Zorba *et al.*, Allosteric modulation of a human protein kinase with monobodies. *Proc Natl Acad Sci U S A* **116**, 13937-13942 (2019).

11. K. Kiianitsa, J. A. Solinger, W. D. Heyer, NADH-coupled microplate photometric assay for kinetic studies of ATP-hydrolyzing enzymes with low and high specific activities. *Anal Biochem* **321**, 266-271 (2003).
